# Prokineticin-1 induces normal lymphangiogenic activity and is involved in lymphangiogenesis and lymph node metastasis in colorectal cancer

**DOI:** 10.18632/oncotarget.28016

**Published:** 2021-07-06

**Authors:** Takayuki Naruse, Takanori Goi, Akio Yamaguchi

**Affiliations:** ^1^First Department of Surgery, University of Fukui, Fukui 9101193, Japan; ^2^Fukui Health Science University, Fukui 9103113, Japan

**Keywords:** prokineticin-1, lymphangiogenic activity, colorectal cancer

## Abstract

Background: Prokineticin family correlates with important roles in several biological processes, including homeostasis. We discovered novel functions of prokineticin1 (PROK1) in lymphangiogenesis and lymphnode metastasis in colorectal cancer.

Materials and Methods: We examined changes in the number of lymphatic endothelial cells after PROK1 stimulation. PROK1 protein was stimulated with subcutaneously implanted in mice. Also a high-PROK1-expressing colorectal cancer cell line and anti-PROK1 antibody(Ab) were subcutaneously implanted in mice, and then examine lymphangiogenesis. PROK1 expression and the number of lymph vessels were examined in the primary lesion of 391 patients whose colorectal tumors had been resected.

Results: When PROK1 was used as a stimulus, the number of lymphatic cells increased compared to unstimulated cells. And the number of lymph vessels in the skin of mice increased compared to mice implanted without PROK1. The number of lymph vessels in the primary tumor tissue increased when PROK1 was highly expressed compared to cases with non-detectable PROK1 expression. When PROK1 was expressed in human colorectal tumors, the rate of lymphnode metastasis was significantly higher than that in cases with non-detectable PROK1 expression.

Conclusions: PROK1 is a lymphangiogenic factor involved in the formation of new lymph vessels and lymphnode metastasis in human colorectal cancer.

## INTRODUCTION

The morbidity rate for colorectal cancer is very high in Japan and in western countries [1–2]. While the use of anticancer and molecular-target drugs for unresectable colorectal cancer has improved its survival rate [3–5], this type of cancer has not yet been eradicated, and therefore, further countermeasures are needed. In colorectal cancer, hematogenous, lymph node, and peritoneal metastases often occur, and are important factors for tumor progression and adequate prognosis [6, 7]. Therefore, elucidation of the mechanisms driving metastasis in colorectal cancer can improve the prognosis of patients and expedite the development of new therapeutic strategies. Lymphangiogenesis is considered an important factor for the development of lymph node metastases. It is known to be promoted by signaling via tyrosine kinase receptors, such as VEGF-C/VEGFR2 (VEGF family), Ang1/Tie2 receptor, FGF2/FGF receptor 3, and HGF/c-Met receptor [8–10].

Prokineticin-1 (PROK1) was cloned by Ferrara, and characterized as an angiogenic growth factor that selectively acts on the endothelium of endocrine cells. The gene encoding PROK1 is positioned at chromosome 1p21, with the coding region consisting of 105 amino acids [11]. The mature protein is formed from 86 amino acids, including 10 cysteines. PROK1 exerts proliferative and migratory activities on endocrine cells, and it promotes the growth of vascular endothelium under hypoxic conditions. However, it does not share homology with the proangiogenic factor VEGF. We previously reported, for the first time, that the increase of PROK1 is relevant to angiogenesis and hepatic metastasis of colorectal cancer cells in patients [12, 13]. Other studies demonstrated that PROK1 expression correlates with malignancy in prostate cancer, neuroblastoma, thyroid cancer, pancreatic duct cancer, gastric cancer, and small intestinal cancer, indicating that PROK1 is important for malignant tumor formation [14–19]. Furthermore, we have previously shown that PROK1 was highly expressed in the LOVO and DLD1 as cell lines [20]. PK-R1 and PK-R2 were identified as PROK1 receptors, and the interaction of PROK1 with its receptors drives various physiological phenomena [21–23]. The signaling pathway activated by this interaction involves conjugation of the PK receptor with Gq, Gi, or Gs proteins, which facilitate the regulation of intracellular calcium dynamics, phosphorylation of p44/p42 MAP kinase, activation of serine-threonine kinase Akt, and cAMP accumulation [24]. These actions may intricately be related to many functions.

In the present study, we observed that PK-R1 and PK-R2 were expressed in normal lymphatic endothelial cells and that proliferation of lymphatic endothelial cells and lymphangiogenesis are induced by PROK1 stimulation. Furthermore, we identified a new relationship between PROK1 expression and lymphatic formation, invasion, and metastasis in human colorectal cancer.

## RESULTS

### Expression of PROK1 receptors (PK-R1 and PK-R2) in human lymphatic endothelial cells

**Figure 1:**
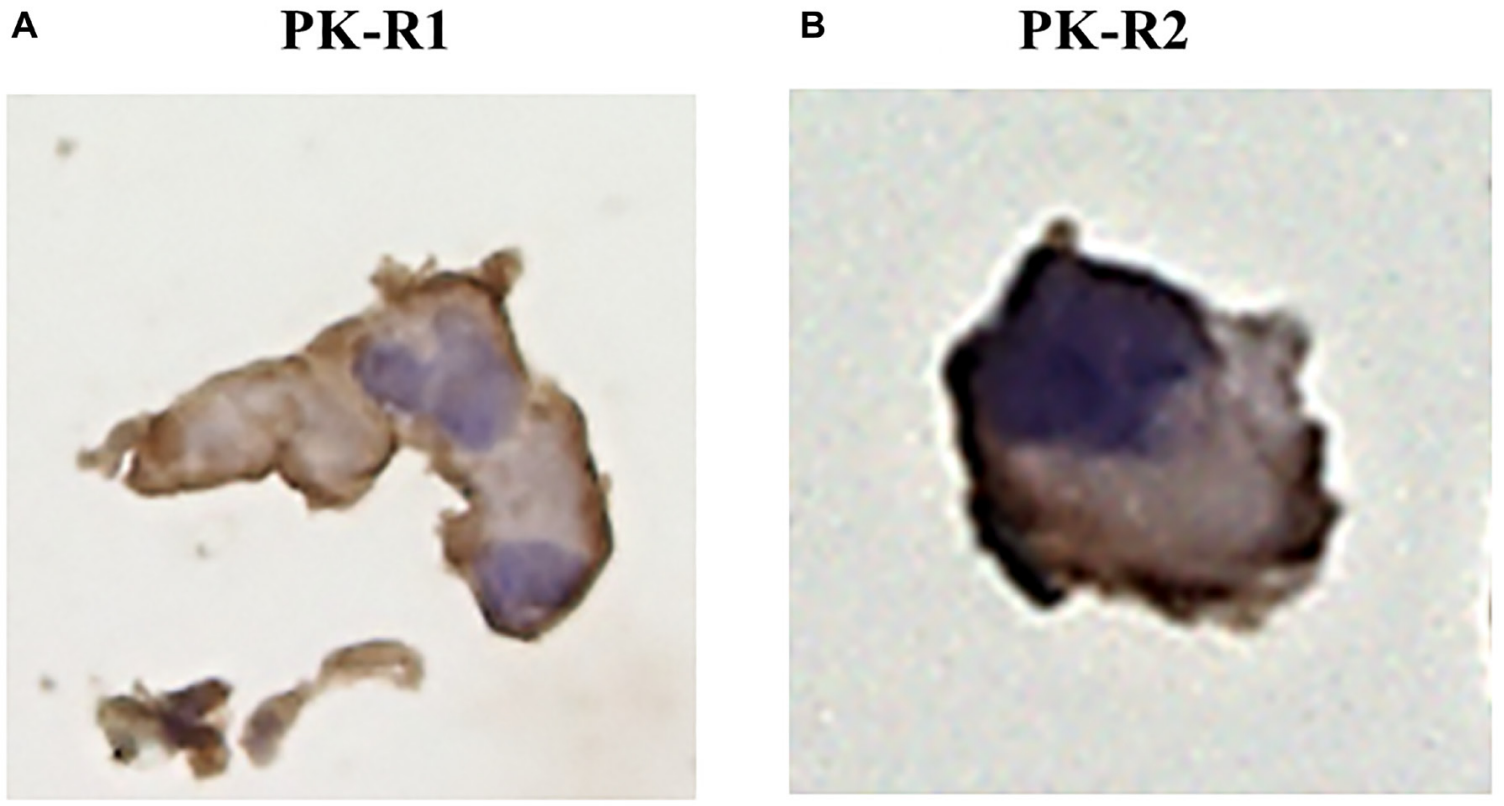
The expression of PK-R proteins in a human lymphatic endothelial cell line by immunohistochemical staining with anti- PK-R1 or PK-R2 mAb. (**A**) PK-R1 expression. (**B**) PK-R2 expression.

Immunohistochemical analyses using anti-PK-R1 and anti-PK-R2 antibodies demonstrated the expression of PK-R1 and PK-R2 in a human lymphatic endothelial cell line (Figure 1).

### Proliferative capacity of human lymphatic endothelial cells

**Figure 2:**
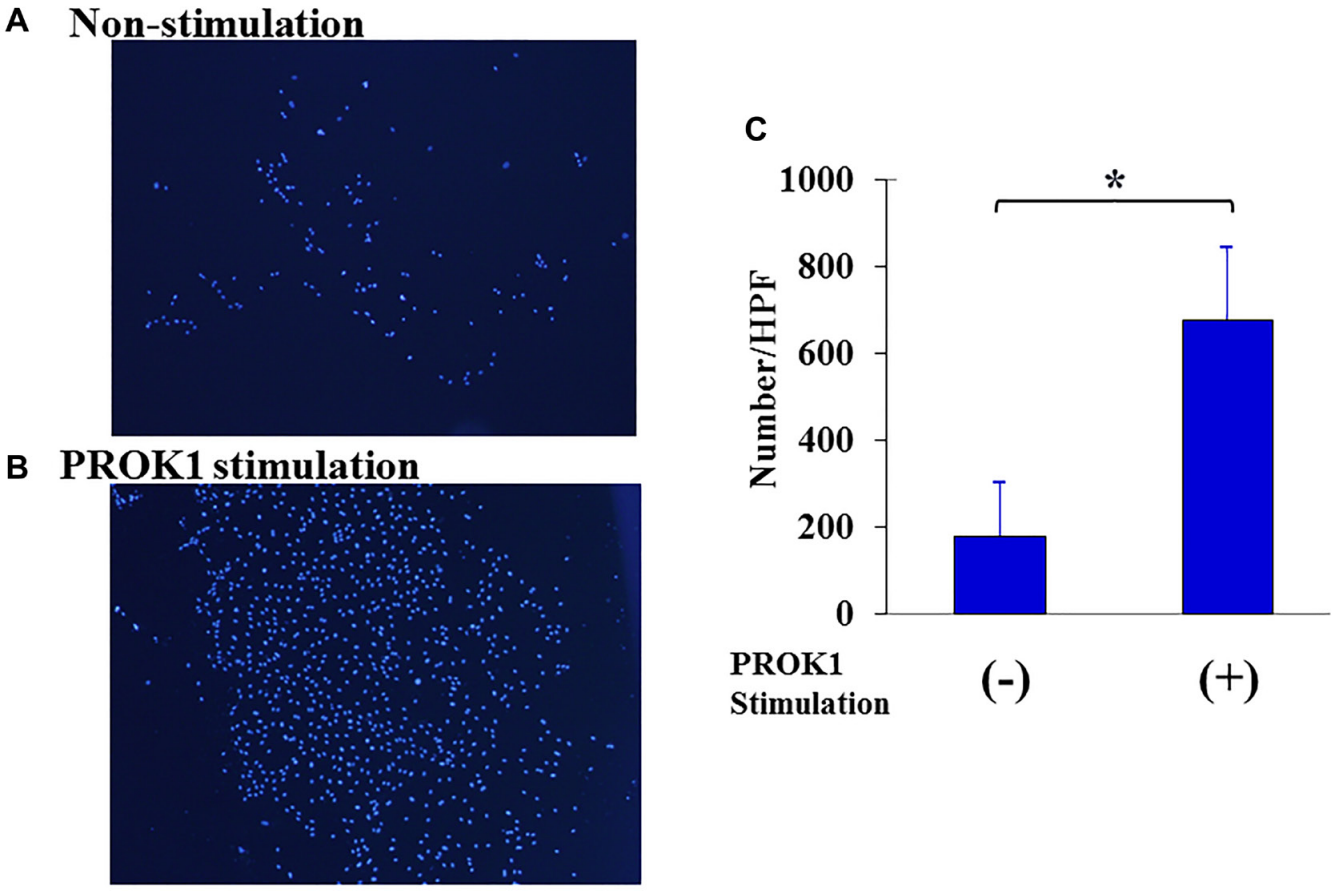
Proliferation of human lymphatic endothelial cells. (**A**) Representative photographs of lymphatic endothelial cells. 1: Non-PROK1 stimulation, (**B**) PROK1 stimulation. (**C**) The numbers/HPF of lymphatic endothelial cells. Data represent means ± SEM. (*n* = 3) (^*^student *t*-test *p* < 0.01).

Figure 2 shows the change in the number of cells when the human lymphatic endothelial cell line was stimulated with PROK1. The number of human lymphatic endothelial cells was 15 per visual field without stimulation; however, the number of cells significantly increased to 225 per visual field with PROK1 stimulation.

### Lymphangiogenesis by PROK1 in mouse skin

**Figure 3:**
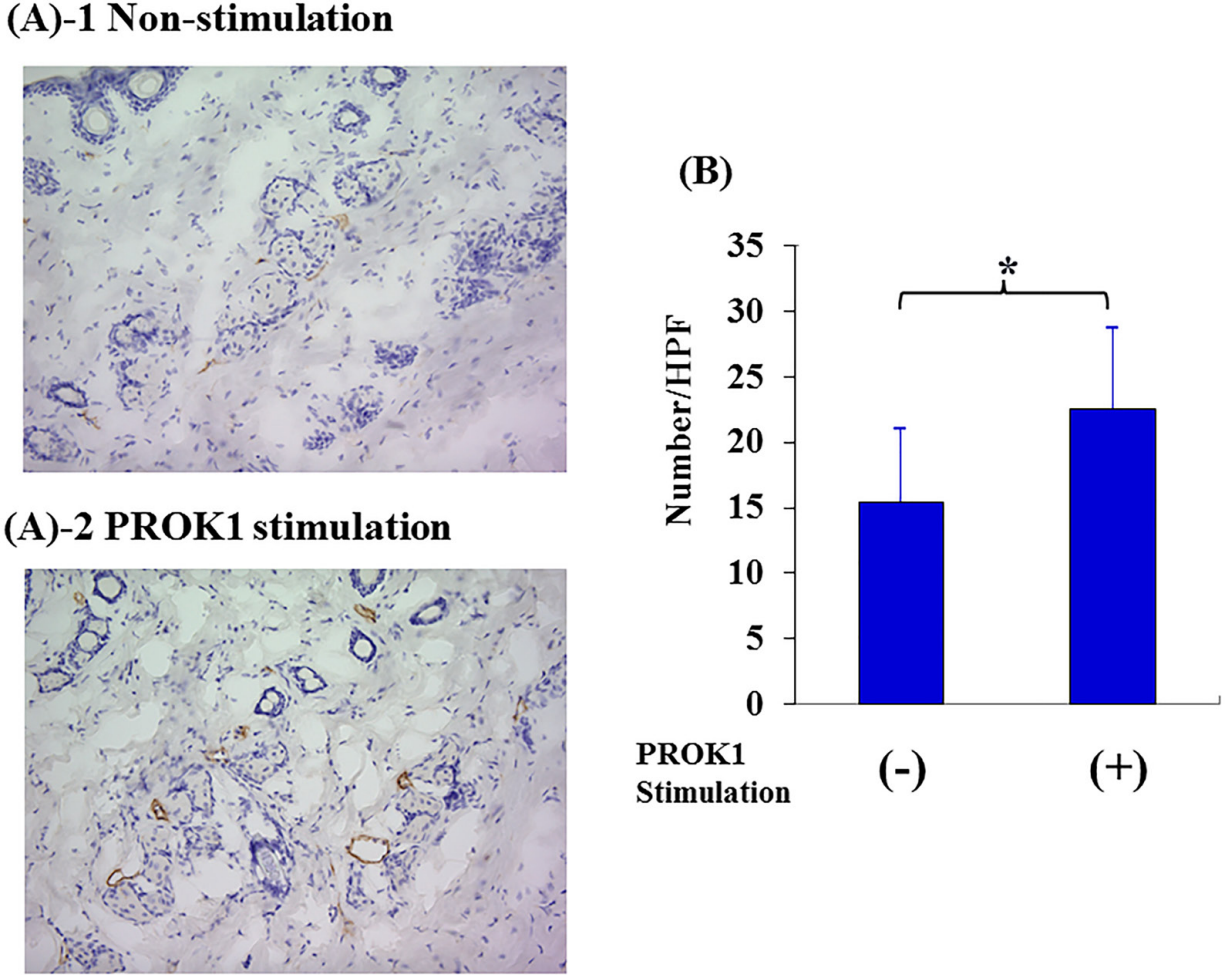
Investigation of Subcutaneous lymphangiogenesis by PROK1 protein. (**A**) Representative photographs of LYVE-1 stained cells. 1: Non-PROK1 stimulation, 2: PROK1 stimulation. (**B**) The numbers of LYVE-1 stained cells. Data represent means ± SEM. (*n* = 3) (^*^student *t*-test *p* < 0.01).

The number of lymph vessels was 12.5 per visual field in the skin tissue adjacent to the subcutaneously implanted chamber containing no PROK1; however, the number of lymph vessels significantly increased to 19.8 per visual field when PROK1 was added to the chamber (Figure 3).

### Lymphangiogenesis in mice subcutaneously injected with colorectal cancer cells highly expressing PROK1 and anti-PROK1 antibody

**Figure 4:**
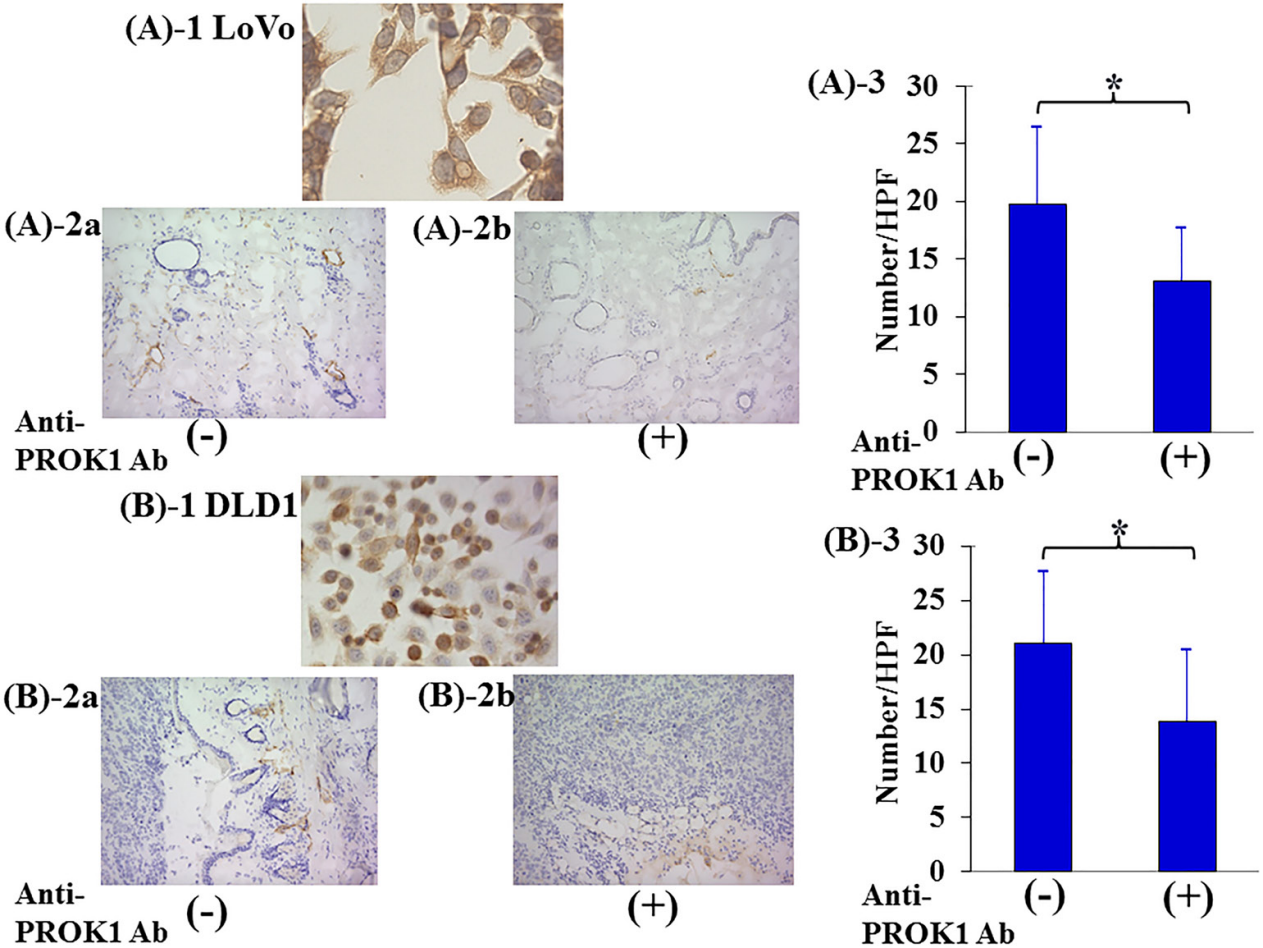
Investigation of Subcutaneous lymphagiogenesis by the anti-PROK1Ab. SHO nude mice were subcutaneously injected in the right armpit region with 1.0 × 10^6^ LoVo or DLD-1 colorectal cancer cells and the anti-PROK1mAb in matrix gel. (A)-1:Representative photographs of PROK1 expression of LoVo colon cancer cells. (A)-2:Representative photographs of LYVE-1 stained cells. (**A**) LoVo cells alone, (**B**) anti-PROK1mAb plus LoVo cells. (A)-3: The numbers/HPF of LYVE-1 stained cells in subcutaneous tumors. Data represent means ± SEM. (*n* = 3) (^*^student *t*-test *p* = 0.01). (B)-1:Representative photographs of PROK1 expression of DLD-1 colon cancer cells. (B)-2:Representative photographs of LYVE-1 stained cells. (A) DLD-1 cells alone, (B) anti-PROK1mAb plus DLD-1 cells. (B)-3:The numbers/HPF of positively LYVE-1 stained cells. Data represent means ± SEM. (*n* = 3) (^*^student *t*-test *p* = 0.03).

The number of lymph vessels was 19.5 per visual field in the skin tissue adjacent to subcutaneously injected high-expressing-PROK1 LoVo colorectal cancer cells. However, the number of lymph vessels decreased to 13 per visual field with the addition of the anti-PROK1 antibody. The number of lymph vessels was 20.5 per visual field in the skin tissue adjacent to subcutaneously injected high-expressing-PROK1 DLD1 colorectal cancer cells, and there were 13.5 per visual field when the anti-PROK1 antibody. The number of lymph vessels was significantly suppressed by the addition of the anti-PROK1 mAb (Figure 4).

### PROK1 expression in the primary lesion of colorectal cancer and the number of lymph vessels in the surrounding tissue

**Figure 5:**
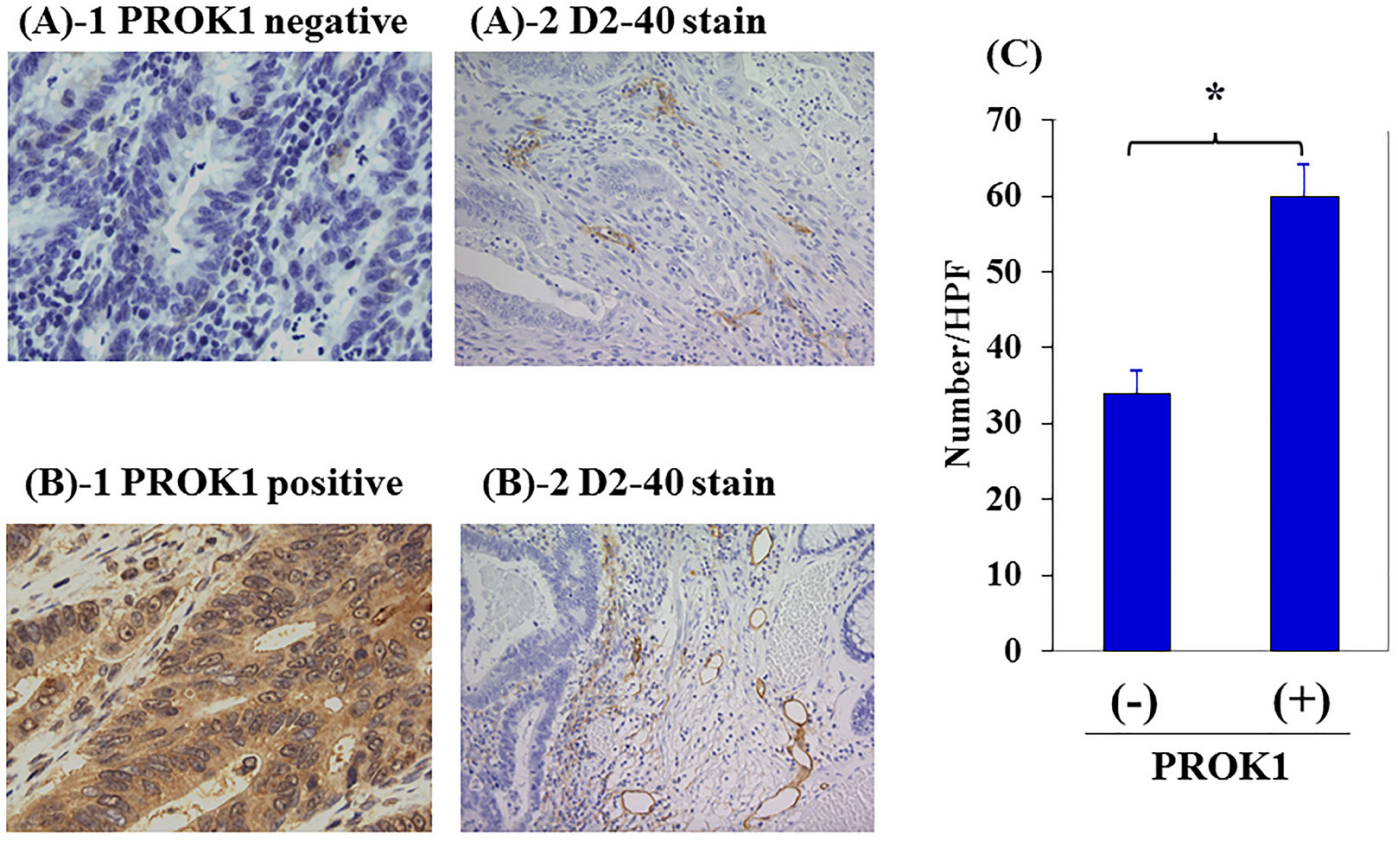
Lymph vessels in human primary colorectal cancer by immunohistochemical staining with anti-D2-40 mAb. (**A**) Representative photographs of D2-40 stained cells in negative PROK1 expression in the primary lesion. 1: PROK1 negative expression, 2: D2-40 expression. (**B**) Representative photographs of D2-40 stained cells in positive PROK1 expression in the primary lesion. 1: PROK1 positive expression, 2: D2-40 expression. (**C**) The numbers/HPF of positively D2-40 stained cells. Left: PROK1 negative case, Right: PROK1 positive case. The numbers/HPF of positively D2-40 stained cells. Data represent means ± SEM. (^*^student *t*-test *p* < 0.01).

PROK1 expression was found in the primary lesion of 142 of 391 (36%) colorectal cancer patients who had undergone resection in our department. Figure 5 shows representative immunohistochemical images of lymph vessels in the surrounding tissue. In cases where PROK1 expression was not observed in the primary colorectal cancer tissue, the number of lymph vessels was 33 per visual field in the surrounding skin tissue, whereas the number of lymph vessels significantly increased to 59.5 per visual field in the surrounding skin tissue of patients with high PROK1 expression, demonstrating lymphangiogenesis (Figure 5).

### PROK1 expression in the primary lesion of human colorectal cancer and lymph node metastasis

Lymph node metastasis was observed in 100 of 249 (40.2%) colorectal cancer patients with negative PROK1 expression in the primary lesion, whereas 96 of 142 (67.6%) patients with positive PROK1 expression, demonstrating a significant increase in lymphatic metastasis with PROK1 expression in the primary lesion (Table 1).

**Table 1:** Lymphnode metastasis according to PROK 1 expression

	Lymphnode Metastasis		
PROK1	All cases	Positive Cases (%)	*P*
Negative	249	100 (40.2%)	
Positive	142	96 (67.6%)	0.003

## DISCUSSION

Under normal healthy conditions, lymph vessels, which are networked throughout the body, play the significant role of drawing tissue fluid leaking from blood vessels and returning the tissue fluid to the blood vessel [25]. However, in cancer, malignant cells invade the lymph vessel, which facilitates migration from the primary lesion to the lymph node, and further spreading to different organs, leading to lymph node metastasis. Therefore, invasion of lymph vessels is the main pathway for metastasis [25–27]. Lymph node metastasis, one of the metastatic types of colorectal cancer, is relevant to the level of tumor malignancy. The presence of lymph node metastases serves as a factor for staging classification and diagnostic consideration [28–32]. Moreover, lymph node metastasis and lymphangiogenesis are presumed to be closely related [33–36]. Accordingly, the discovery of a regulating factor for the formation of lymph vessels will be important for elucidating the cause of lymph vessel-related problems, including benign edema and lymph node tumor metastasis.

Lymphangiogenesis is a sequence of lymph vessel formation initiating from migration and proliferation of lymphatic endothelial cells. VEGF-C, PDGF-BB, FGF-2, angiopoietin-1, HGF, and other angiogenesis-related proteins have been reported as factors for lymphangiogenic activity [8, 9, 37, 38], and they may act on lymphatic endothelial cells to promote the formation of new lymph vessels. The VEGF family proteins (which includes VEGF-C) are well-known factors involved in colorectal cancer and other malignant tumors, and they form multiple proteins by protein splicing [39]. VEGF-A, in particular, is a known angiogenic factor, and the chemotherapeutic drug targeting it has demonstrated a substantial level of efficacy in cancer treatment [40]. Furthermore, VEGF-C is produced in tumors and promotes lymphangiogenesis via the Flt4 receptor, which may induce the formation of new lymph vessels and the metastasis of tumors to the regional lymph node and distant organs [41]. VEGF-C is therefore considered a risk factor for a poor prognosis in tumor patients. In the pancreatic islet tumor mouse model, lymphangiogenesis was induced in pancreatic islet tumors, following the expression of VEGF-C in pancreatic beta cells [42]. However, lymph node metastasis was suppressed by inhibition of lymphangiogenesis via neutralizing antibody-mediated inhibition of its receptor, VEGFR3 [43]. Based on these findings, lymph vessels are considered important for malignant tumors. PROK1, investigated in the present study, was cloned by Ferrara, and it was characterized as an angiogenic growth factor that selectively acts on the endothelium of endocrine gland cells [11]. It is different from VEGF, despite their similar functions. We report here that PROK1 is involved in angiogenesis and hematogenous metastasis, including hepatic metastasis, in colorectal cancer. Recent studies demonstrate the expression of PROK1 in digestive tract cancer (gastric, small intestine, etc.), as well as in neuroblastoma, thyroid cancer, and pancreatic duct cancer [14–19]. These findings highlight its relationship with malignant tumors. In the present study, we first confirmed the expression of PROK1 receptors in normal lymph vessel cells. Upon stimulation with PROK1, the proliferative capacity of lymph vessel cells was enhanced, and PROK1 was confirmed to be a lymphatic growth factor. The high-PROK1-expressing colorectal cancer cell line was found to extracellularly secrete PROK1, and lymphangiogenesis was suppressed using the anti-PROK1-antibody. These findings indicate the significance of PROK1 in malignant tumors and its possible development as a new therapeutic target.

The tumor microenvironment has recently been considered to be significant for growth and progression of tumor cells. It consists of fibroblasts, inflammatory cells, immunocompetent cells, blood vessels, and lymph vessels, each of which plays important roles in tumor development [44–47]. The findings of the present study show that PROK1 expression and growth of lymphatic endothelial cells are correlated, and the incidence of lymph node metastasis is greater in patients with PROK1 expression in the primary tumor lesion, compared to those with non-detectable PROK1 expression in human colorectal cancer. This indicates that in colorectal cancer, the tumor itself organizes the surrounding environment via PROK1 expression, which plays an important role within the tumor microenvironment. Colorectal cancer with high lymph vessel invasion tends to have high lymph node metastasis. Since PROK1 induces lymphangiogenesis, it is expected to suppress lymph node recurrence by suppressing the expression of PROK1.

The role of PROK1 in the tumor microenvironment has been revealed by the present study, which leads the development of a new therapy for colorectal cancer by providing basic insight for the molecular mechanism of invasion and metastasis.

## MATERIALS AND METHODS

### Antibody (Ab)

The antibodies were used: anti-mice LYVE-1 Ab (Medical & Biological Laboratories Co., Ltd., Japan), anti-human D2-40 Ab (Nichirei Corporation, Japan), anti-human PK-R1 Ab (Novus Biochemicals Co., USA) and anti-human PK-R2 Ab (Novus Biochemicals Co., USA), anti-human PROK1 Ab (prepared in our department) [48].

### Cell culture (colon cancer cell lines)

The human colon cancer cell lines, DLD-1 and LoVo (American Type Culture Collection. ATCC) were cultured in RPMI1640 medium supplemented with 10% fetal bovine serum (FBS), 100 U/mL streptomycin and 100 U/mL penicillin (Gibco/Invitrogen, USA) at 37°C in 5% CO2 [49]. The authors and were passed less than 10 times since obtaining the cells.

### Cell culture (lymphatic microvascular endothelial cell line)

The human Dermal Lymphatic Microvascular Endothelial Cells (Lonza, Japan) were cultured in EGM™-2MV Microvascular Endothelial Cell Growth Medium-2 BulletKit™ (CC3202) medium (Lonza, Japan).

### Cell growth

The lymphatic vessel cells (1.0 × 10^2^ cells) were seeded onto a well (96 well plate), and were incubated with PROK1 protein (20 ng/ml) at 37°C for 72 hours. The nucleus of cells were stained by using DAPI (diamidino-2-phenylindole) (Sigma-Aldrich. USA). The cell number were counted at magnification 200×.

### Cell culture fluid

Each cell line was passaged at 60% confluence in a 60-mm culture dish, and cultured in RPMI1640 containing 10% FBS for 3 days. The culture fluid was collected after culture of the cell lines.

### Detection of vascularization with Dorsal air sac method

A Millipore chamber (Millipore; diameter, 10 mm: filter pore size, 0.45 μm) was filled with Phosphate buffered saline (PBS) plus PROK1 protein was implanted under the skin into the dorsal side of six-week-old female SHO nude mice (Charles River, Japan). At 7 days after implantation, a rectangular incision was made in the skin on the dorsal side [49].

### Lymphatic vessel formation in nude mice

Six-week-old female SHO nude mice (Charles River, Japan) were subcutaneously injected in the the dorsal side with 1.0 × 10^6^ cells in 0.1 mL of matrix gel (BD Biosciences, USA). Two groups of mice were tested. Group A was injected with non-stimulated colon cancer cells (DLD-1 and LoVo) and normal mouse IgG. Group B was injected with colon cancer cells and the anti-PROK1 mAb (5 μg). After 21 days after implantation, the tumor resection was made in the skin on the dorsal side. The skin was embedded in OCT compound (Sakura Finetechnical, Japan) [50]. Four-μm-thick sections were analyzed for lymphatic cells the expression of LYVE-1 protein by the ChemMate method using the EnVision system (DAKO). For lymphatic vessel counting, one field magnified 200-fold in each of five vascularized areas was counted, and average counts were recorded [49].

### Patients and samples

Primary colorectal cancer tissues and adjacent normal colorectal tissues were obtained from surgical resection from 391 patients with sporadic primary colorectal cancer in the First Department of Surgery, University of Fukui, Japan between 1990 and 2007. According to the TMN classification [6], 76, 109, 135, and 71 were I, II, III, and IV respectively. As histopathological findings varied within the same tumors, the diagnosis was based upon the dominant pattern evaluated by two pathologists [7]. All sample were fixed in 10% paraformaldehyde (pH6.8) for 24 h, and embedded in paraffin.

The eligibility criteria were as follows: (1) a histopathological findings confirmed primary colorectal cancer; (2) resection of colorectal cancer with extended (D2 or D3) lymphnode dissection [7]; (3) histological curative resection (Stage I~III); (4) no chemotherapy or radiotherapy before surgical resection [51].

### Immunohistochemical study

Paraffin sections (4 μm thick) were deparaffinized with xylene and dehydrate through a graded ethanol series. Endogenous peroxidase activity was blocked by incubation for 30 minutes with 1% hydrogen peroxidase in methanol. These hydrate sections were incubated in a dilution of normal goat serum at room temperature for 20 minutes to reduce nonspecific staining, and incubated with anti-PROK1 mAb or anti-D2-40 Ab for 1 hour. After washing with TBS, and analyzed for the expression of or D2-40 protein by the ChemMate method using the EnVision system (DAKO, Danmark). Finally, the slides were lightly counterstained with hematoxylin. The expression was interpreted as positive when the protein was expressed in more than 30% of all cancer cells.

### Statistical analysis

Statistical significance was determined by student *t*-test or the χ2 test using Stat Mate IV (ATMS Co., Ltd., Japan). *P* value of < 0.05 were considered statistically significant.

## CONCLUSIONS

To our knowledge, the present study was the first to identify PROK1 as a lymphangiogenic factor, and the molecular mechanism of the process from lymphangiogenesis through to lymph node metastasis was newly discovered in human colorectal cancer. Our findings indicate the potential of development of a new therapy.
